# *BRAF*V600E Mutation Analysis in Patients with Metastatic Colorectal Cancer (mCRC) in Daily Clinical Practice: Correlations with Clinical Characteristics, and Its Impact on Patients’ Outcome

**DOI:** 10.1371/journal.pone.0084604

**Published:** 2013-12-18

**Authors:** Zacharenia Saridaki, Maria Tzardi, Maria Sfakianaki, Chara Papadaki, Alexandra Voutsina, Aristea Kalykaki, Ippokratis Messaritakis, Kyriakos Mpananis, Dimitris Mavroudis, Efstathios Stathopoulos, Vassilis Georgoulias, John Souglakos

**Affiliations:** 1 Laboratory of Tumor Cell Biology, School of Medicine, University of Crete, Heraklion, Crete, Greece; 2 Department of Medical Oncology, University General Hospital, Heraklion, Crete, Greece; 3 Laboratory of Pathology, University General Hospital, Heraklion, Crete, Greece; University de Minho, Portugal

## Abstract

**Background:**

To prospectively evaluate the usefulness of the *BRAF*V600E mutation detection in daily clinical practice in patients with metastatic Colorectal Cancer (mCRC).

**Patients and Methods:**

504 mCRC patients treated with systemic chemotherapy ± biologics were analyzed.

**Results:**

A statistically significant higher incidence of the BRAF mutation was observed in patients with ECOG-PS 2 (*p*=0.001), multiple metastatic sites (*p*=0.002),> 65 years old (*p*=0.004), primary tumors located in the colon (*p*<0.001), high-grade tumors (*p*=0.001) and in those with mucinous features (*p*=0.037). Patients with *BRAF*V600E mutated tumors had a statistically significantly reduced progression-free survival (PFS) compared to wild-type (wt) ones (4.1 and 11.6 months, respectively; *p*<0.001) and overall survival (OS) (14.0 vs. 34.6 months, respectively; *p*<0.001). In the multivariate analysis the *BRAF*V600E mutation emerged as an independent factor associated with reduced PFS (HR: 4.1, 95% CI 2.7–6.2; *p*<0.001) and OS (HR: 5.9, 95% CI 3.7–9.5; *p*<0.001). Among the 273 patients treated with salvage cetuximab or panitumumab, the *BRAF*V600E mutation was correlated with reduced PFS (2.2 vs. 6.0 months; *p*<0.0001) and OS (4.3 vs. 17.4 months; *p*<0.0001).

**Conclusions:**

The presence of *BRAF*V600E-mutation in mCRC characterizes a subgroup of patients with distinct biologic, clinical and pathological features and is associated with very poor patients’ prognosis.

## Introduction

 Mutations in the *BRAF* oncogene have been found in approximately 8% of human cancers, including 50-60% of melanomas, 30-70% of thyroid cancers, 30% of serous low-grade ovarian cancers and 10% of CRCs[1]. The most common oncogenic mutation accounting for more than 95% of the mutations in *BRAF* found in CRCs is the single substitution missense mutation V600E, which is located within the kinase domain of the gene[2]. This amino acid change results in constitutive activation of the BRAF kinase and promotes cell transformation[1,3].. Mutations in other codons of the *BRAF* gene in colon cancer are extremely rare, counting for <5% of all mutations in the gene [2].

Several studies have reported that the existence of a *BRAF* mut ation in a primary CRC tumor marks patients who carry an especially poor prognosis, regardless o ftreatment type administration. Its presence has been associated with decreased survival in early-operable stages treated with adjuvant chemotherapy[4]. similarly, in the metastatic disease setting patients do not seem to respond to any of the existing chemotherapy regimens and their outcome resembles that of untreated patients[5-8]. In CRC, *BRAF* mutations are reported to occur more frequently in cases characterized by the presence of a defective DNA mismatch repair (dMMR) system resulting in microsatellite instability (MSI)[9-11]; this seems to be due to hMLH1 promoter hypermethylation (sporadic CRC) and not to germ-line alterations (hereditary CRC)[12,13]. As it has been previously reported, the *BRAF* mutation retains its prognostic value both in MSI-high and in microsatellite stable (MSS) tumors[4-6,14]; the latter being also confirmed by the recently published *BRAF* signature[15].

Besides its prognostic implications, several retrospective studies have attributed a predictive role to the *BRAF*V600E mutation due to the observed lack of benefit related to treatment with anti-EGFR moAbs. This was, initially, first documented by Di Nicolantonio et al[3], and Souglakos et al[8], but over the years, this was further confirmed by subsequent studies[7,16,17]. Furthermore, this mutation’s adverse prognostic significance was confirmed in the post-hoc subgroup analysis in two first line phase III randomized trials [CAIRO2 and CRYSTAL][18,19]. Despite the fact that, the above mentioned data require further validation in prospective randomized trials, they support the notion that the natural history and response to treatment to various chemotherapeutic regimens of *BRAF*-mutant CRC tumors differ markedly from the *BRAF* wild type tumors. Apparently, a mutant *BRAF* does not simply substitute for *KRAS* activation in a linear signaling pathway; most likely it confers distinct characteristics with ominous consequences, something which justifies its utilization in patient selection and stratification in future clinical trials[8].

In order to evaluate the usefulness of the *BRAF*V600E mutation detection in daily clinical practice, to investigate its correlation with the various clinico-pathological characteristics, as well as, its prognostic and predictive impact, we sought to conduct this study in a prospective database of CRC patients treated for metastatic disease. 

## Patients and Methods

### Patient population

The study was approved by the Ethics Committee/ Institutional review board of the University Hospital of Heraklion and all patients gave their written informed consent for the use of the tissue material for translational research. Since 1/1/2007 until 31/12/2012, we prospectively analyzed for *BRAF* V600E all patients with newly diagnosed mCRC at the Department of Medical Oncology, University Hospital of Heraklion (Crete, Greece). Five hundred and four consecutive patients, with histologically confirmed mCRC and available tumor material for molecular analysis, who were treated with at least one cycle of systemic chemotherapy with or without the addition of bevacizumab, cetuximab or panitumumab were enrolled. Patients’ evaluation was performed at baseline and every four cycles of chemotherapy. Disease status was coded, without the knowledge of the laboratory analysis. 

### Tissue selection and DNA extraction

Formalin-fixed, paraffin-embedded (FFPE) tumor sections were reviewed by a pathologist (MT) to confirm the diagnosis and define tumor-enriched areas for dissection. Ten serial sections of 5μm thickness were stained with nuclear fast red (Sigma-Aldrich, St Louis, MO, USA) and scrape dissection under a binocular microscope was performed for samples with ≥ 80% tumor cells; for samples with < 80% malignant cells, microdissection with the piezoelectric Eppendorf microdissector (Eppendorf, Hamburg, Germany) was performed. DNA extraction was performed using the MasterPure™ Complete DNA and RNA Purification Kit according to the manufacturer’s instructions (Epicentre Biotechnologies, Madison, WI, USA) and the isolated cancer cells were lysed in buffer containing Proteinase K at 60 °C for 72 h.[11] 

### KRAS mutational analysis

KRAS mutational analysis was performed by Sanger sequencing after PCR amplification of KRAS exon 2. PCR conditions and primers sets used have been previously reported[8]. 

### BRAF mutational analysis

The V600E BRAF mutation was detected by real-time PCR using the allelic discrimination method as previously described[11,20]. In brief, tumor cells’ DNA was amplified with the use of a set of primers and two hydrolysis probes in the ABI PRISM 7900T Sequence Detection System (AB; Applied Biosystems, Forest City; CA; USA). The two hydrolysis probes were labeled at 5’ with VIC and FAM fluorophores reporters for the wt and the mutant allele, respectively. The SDS 2.3 software was used for the analysis of the results.

### Study Design

The aim of this study was to evaluate the usefulness of the *BRAF*V600E detection in the daily clinical practice and to correlate its existence with clinical and pathological characteristics, as well as treatment outcome in order to define possible prognostic and/or predictive implementations in a prospective database of patients with mCRC. All available biopsies of the primary tumor with more than 100 cells per section were included in the analysis. Associations between *BRAF* and baseline characteristics were assessed using the Fisher's exact test for categorical variables or logistic regression for continuous variables. Progression Free Survival (PFS) and overall survival (OS) were measured from the date of diagnosis of metastatic disease to the first radiographic documentation of disease progression or death, respectively. Kaplan–Meier curves were used to describe the proportion of patients who remained free of events over the follow-up period. Associations between prognostic factors and PFS or OS were examined using Cox proportional hazards regression models. All reported p-values are two-sided and not adjusted for multiple testing.

## Results

### Patients’ characteristics and disease features

The characteristics of the enrolled patients were typical for metastatic CRC and are summarized in Table 1. In brief, the median patients’ age was 64 year (range: 21-89), 59% were men and their PS (ECOG) was 0-1 (90%); the primary tumor was located in the rectum in 28% of the patients and in 40% of the cases was undifferentiated (high grade) (Table 1). Twenty-seven per cent of the patients had one metastatic site and 64 (13%) underwent a metastasectomy with curative intent after the administration of systemic treatment. The *BRAF*V600E mutation was detected in 41 (8.2%) patients and in all cases was mutually exclusive with *KRAS* mutations which were detected in 217 (43%) of the total study population. 

**Table 1:** Patients’ and Tumors’ Characteristics and Univariate analysis of Survival.

**Feature**	**Ν**	**%**	**Progression Free Survival**				**Overall Surivival**			
	504	100	Median (months)	HR*	95% CI**^*@*^**	*p* value	Median (months)	HR*	95% CI**^*@*^**	*p* value
Median Age (Range)	64(21-89)									
**Age**										
≤ 65 years	271	54	11.5	1.17	0.91-1.51	0.225	32.3	0.82	0.61-1.04	0.07
> 65 years	231	46	10.0				27.8			
**Gender**										
Male	297	59	9.9	1.11	0.86-1.43	0.42	33.2	0.98	0.76-1.25	0.85
Female	207	41	11.2				34.6			
**Tumor Location**										
Colon	362	72	10.7	1.05	0.72-1.52	0.802	33.8	0.96	0.71-1.71	0.65
Rectum	142	28	11.1				33.7			
**Tumor Differentiation**										
High Grade (Undifferentiated)	200	40	7.9	1.81	1.40-2.34	0.001	23.8	2.29	1.74-3.02	0.001
Low Grade (Well-Moderate Differentiated)	304	60	11.6				34.9			
**MucinousFeatures**										
Yes	99	20	9.8	1.27	0.68-2.38	0.089	29.8	1.42	1.18-2.01	0.124
No	405	80	11.2				33.5			
**ECOG PS^*#*^**										
0-1	454	90	11.9	1.96	1.54-2.83	0.001	35.7	2.31	1.18-4.06	0.027
2	50	10	7.8				17.5			
**Number of metastatic sites**										
**1**	139	27	11.4	1.24	0.84-1.82	0.95	33.8	1.58	0.91-2.13	0.097
**>1**	365	73	9.8				28.9			
**Metastasectomy**										
Yes	65	13	22.3	0.29	0.09-0.42	<0.001	52.7	0.31	0.19-0.50	0.001
No	439	87	9.8				29.9			
***KRAS* mutations**										
Wild Type	286	57	10.3	1.06	0.81-1.37	0.69	34.4	1.23	0.90-1.67	0.189
Mutant	218	43	10.9				27.6			
***BRAF*^*V600E*^ mutations**										
Wild Type	462	91.8	11.6	4.07	2.66-6.20	<0.001	34.6	5.43	3.60-8.18	<0.001
Mutant	42	8.2	4.1				14.0			
*KRAS/BRAF* ^*V600E*^ mutations										
Double Wild Type	244	48	13.3	1.89	1.65-2.18	0.034	36.2	2.28	1.79-3.01	0.016
*KRAS* or *BRAF* ^*V600E*^ mutant	260	52	9.6				21.6			

*HR: Hazard Ratio, **^*@*^**CI: Confidence Interval, **^*#*^**PS: Performance Status

### Systemic treatment and patients’ outcome

The median time from initial diagnosis to diagnosis of metastatic disease was 21.6 months (95% CI 17.6–24.2) for patients with early-stage disease (stage I-III) and the median interval from the diagnosis of metastatic disease to treatment initiation 0.6 months (95% CI 0.4–1.0). The median follow up time was 30.4 months (range, 2.6-72.9 months) and at the time of analysis 329 (65%) patients were deceased, mainly from disease progression (n=322; 98%); five (1%) deaths were treatment-related and two (0.4%) were due to reasons unrelated to disease or treatment. The median PFS was 10.5 months (95% CI: 8.9-12.4) and the median OS 29.9 months (95% CI: 26.8-34.5). All patients were treated with 5-FU-based first-line chemotherapy and in 96% of the cases the patients received an oxaliplatin or irinotecan combination (Table 2). Two hundred and thirty nine (48%) patients received also bevacizumab in combination with chemotherapy in the first line setting, while an anti-EGFR monoclonal antibody was administered in 74 (14.7%) of the patients in the first line setting and in 273 (54%) in the 2^nd^ or subsequent treatment lines (Table 2). The vast majority of the patients has been treated with 2^nd^ line systemic treatment. No difference was observed in the percentage of patients treated with 2^nd^ line treatment between *BRAF* WT and mutant patients; groups (*p*=0.314) (Table 3). 

**Table 2:** Systemic treatment.

**REGIMENS**	**N**	**%**
Oxaliplatin-based1^st^ line	174	34
Irinotecan-based 1^st^ line	195	39
FOLFOXIRI	115	23
Fluoropyrimidins monotherapy	18	4
Bevacizumab + chemotherapy 1^st^ line	239	48
Cetuximab or Panitumumab 1^st^ line	74	15
Cetuximab or Panitumumab Salvage treatment	273	54

**Table 3:** Correlation of *BRAF*V600E mutation with clinical and pathological characteristics.

**Feature No (%)**			***BRAF^V600E^***		***p* value**
		Total	Wild Type	Mutant	
**Age**	≤ 65 years	270 (54)	254 (94.1)	16 (5.9)	0.004
	> 65 years	232 (46)	207 (89.2)	25 (10.8)	
**Tumor Differentiation**	Low grade	303 (60)	290 (95.7)	13 (4.3)	0.001
	High grade	199 (40)	171 (86.9)	28 (14.1)	
**Tumor Location**	Colon	361 (72)	324 (89.8)	37 (10.2)	<0.001
	Rectum	141 (28)	137 (97.2)	4 (2.8)	
**Mucinous**	Yes	98 (20)	84 (86)	14 (14)	0.0037
	No	404 (80)	377 (94.6)	27 (5.4)	
**ECO PS^*#*^**	0-1	453 (90)	432 (95.2)	21 (4.6)	<0.001
	2	49 (10)	29 (39)	20 (41)	
**Number of metastatic sites**	1	138 (27)	133 (96.4)	5 (3.6)	0.002
	>1	364 (73)	328 (90.1)	36 (9.9)	
**2^nd^ line treatments**	Yes	489	449 (90.1)	40 (9.1)	0.314
	No	25	23 (86.7)	2 (13.3)	
**Metastasectomy**	Yes	65 (13)	64 (98.5)	1 (1.5)	<0.001
	No	439 (87)	398 (91)	41 (9)	

### Correlations of BRAF mutation with clinico-pathological features and patients’ Progression Free and Overall Survival

The detection of the *BRAF*V600E mutation has been correlated with specific clinical characteristics and pathological features (Table 3). More precisely, the *BRAF*V600E mutation was detected in 10.8% and 5.9% (*p*=0.004) of the patients older and younger than 65 years old, respectively. Also, high grade tumors presented a higher frequency of the *BRAF*V600E mutation (14.1%) in comparison with low grade tumors (4.3%; *p*=0.001). In addition, a higher incidence of the *BRAF*V600E mutation was found in tumors located in the colon (10.2%) than in the rectum (2.8%; *p*<0.001), as well as in tumors with mucinous histology (14%) compared to those with non mucinous features (5.4%; *p*=0.037). Finally, the *BRAF*V600E mutation was more frequently detected in patients with ECOG PS 2 (41%) compared to those with PS 0-1 (4.6%; *p*<0.001) and in patients with multiple metastasis (9.9%) compared to those with one metastatic site (3.6%; *p*=0.002) (Table 3). There was no significant correlation between the *BRAF*V600E mutation status and the gender (*p*=0.412). Only one patient (1.5) with *BRAF* ^*V600E*^ mutation underwent a metastasectomy in comparison with 63 (13%) patients with WT *BRAF* tumors (*p*<0.001).

**Figure 1:**
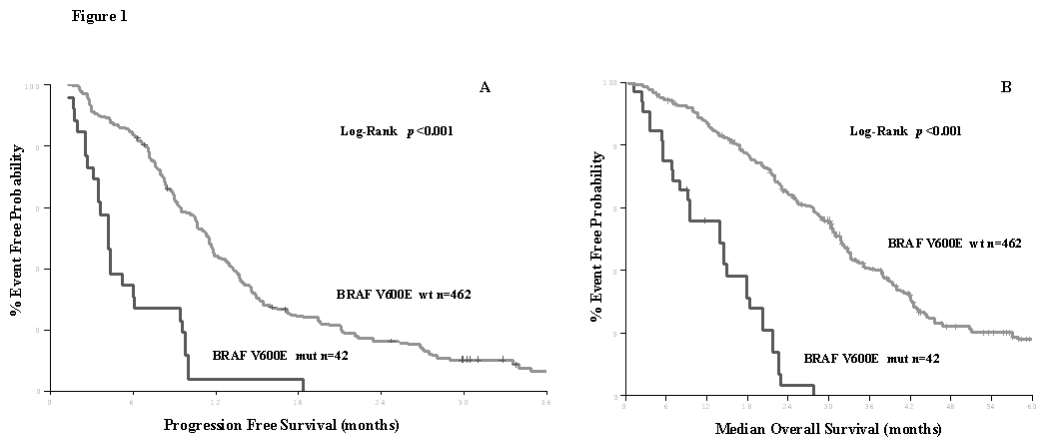
Progression Free Survival in 1^st^ systemic treatment according to BRAF^*V600E*^mutation in 504 patients with metastatic Colorectal Cancer (A) and Median Overall Survival according to BRAF^*V600E*^mutation in 504 patients with metastatic Colorectal Cancer (B).

Univariate analysis revealed significant association of several clinical and pathological features with PFS and/or mOS. Indeed, patients with *BRAF*V600E mutated primary tumors presented significantly lower PFS (4.1 vs. 11.6 months; HR: 4.07, 95% CI: 2.66-6.20; *p*<0.001) in comparison with those with *BRAF* wild type primary tumors (Table 1 and Figure 1A); this finding was independent of the type of the administered first line treatment (all *p* values > 0.05). Similarly, the PFS was significantly lower in patients with high grade tumors (7.9 vs. 11.6 months; HR: 1.81, 95% CI: 1.40-2.34; *p*=0.001) and ECOG PS 2 (7.8 vs. 11.9 months; HR: 1.81, 95% CI: 1.54-2.83; *p*=0.001) in comparison to those with low grade tumors and ECOG PS 0-1, respectively (Table 1). In addition, patients with both KRAS/BRAF^V600E^ WT tumors present significantly higher PFS (13.3 vs. 9.6 months; HR: 1.89, 95% CI: 1.65-2.16; *p*=0.034) in comparison with those with any mutation in *KRAS or BRAF* ^*V600E*^ (Table 1) In contrast, patients who underwent a metastasectomy of a metastasis with curative intent presented significantly higher PFS (22.3 vs. 9.8 months; HR: 0.29, 95% CI: 0.09-0.42; *p*<0.001) (Table 1). There was no significant association between PFS and age, gender, tumor location, mucinous histology, number of metastatic sites and *KRAS* mutations (Table 1).

Univariate analysis also showed that patients with *BRAF*V600E mutated primary tumors had significantly lower median OS (14.0 vs. 34.6 months; HR: 5.43, 95% CI: 3.60-8.18; *p*<0.001) in comparison with those with wt primary tumors (Table 1, Figure 1B), independently from the type of the administered first line treatment. In addition, the median overall survival was significantly higher in patients with low grade tumors (34.9 vs. 23.8 months; HR: 2.29, 95% CI: 1.74-3.02; *p*=0.001), PS 0-1 (35.7 vs 17.8 months; HR: 2.31, 95% CI: 1.18-4.06; *p*=0.001) and those who underwent metastasectomy (52.7 vs. 29.9 months; HR: 0.31, 95% CI: 0.19-0.50; *p*=0.001) (Table 1). Also, patients with both KRAS/BRAF^V600E^ WT tumors present significantly prolonged median OS (36.2 vs. 21.6 months; HR: 2.28, 95% CI: 1.79-3.01; *p*=0.034) in comparison with those with any mutation in *KRAS or BRAF* ^*V600E*^ (Table 1). There was no significant association between mOS and gender, tumor location, mucinous histology, number of metastatic sites or *KRAS* mutations status, while a non significant trend has been observed for improved survival in patients younger than 65 years of age (32.3 vs. 27.8 months; HR: 0.82, 95% CI: 0.61-1.04; *p*=0.07) compared to those over 65 years of age (Table 1).

The multivariate analysis confirmed that the detection of the *BRAF*V600E mutation was strongly correlated with both decreased PFS (HR: 4.1, 95% CI: 2.7-6.2; *p*<0.001) and OS (HR: 5.9, 95% CI: 3.7-9.5; *p*<0.001) (Table 4). Likewise, high grade tumor and poor PS (ECOG 2) emerged as independent prognostic factors for shorter PFS (HR: 2.3, 95% CI: 1.4-3.1; *p*=0.008 and HR: 1.6, 95% CI: 1.2-2.1, respectively; *p*=0.034) and shorter mOS (HR: 2.8, 95% CI: 1.8-4.1; *p*=0.003 and HR: 1.7, 95% CI: 1.4-2.3, respectively; *p*=0.021) (Table 4). Similarly, metastasectomy with curative intent emerged as an independent prognostic factor for improved PFS (HR: 0.4, 95% CI: 0.26-0.8; *p*=0.003) and mOS (HR: 0.6, 95% CI: 0.4-0.9; *p*=0.028) (Table 4).

**Table 4:** Multivariate analysis.

	**Hazard Ratio**	95% CI*	***p* value**
**Progression-Free Survival**			
BRAF (mutant vs. WT*)	4.1	(2.7 -6.2)	<0.001
**Tumor Grade (High vs. Low**)	2.3	(1.4 - 3.1)	0.008
**Metastatectomy (yes vs. no**)	0.4	(0.26- 0.8)	0.003
**ECOG PS (2 vs. 0-1)**	1.6	(1.2-2.1)	0.034
**Overall Survival**			
BRAF (mutant vs. WT*)	5.9	(3.7 -9.5)	<0.001
**Tumor Grade (High vs. Low**)	2.8	(1.8- 4.1)	0.003
**Metastatectomy (yes vs. no**)	0.6	(0.4 - 0.9)	0.028
**ECOG PS (2 vs. 0-1)**	1.7	(1.4-2.3)	0.021

### Predictive significance of BRAFV600E mutation in treatment with anti-EGFR monoclonal antibodies

**Figure 2:**
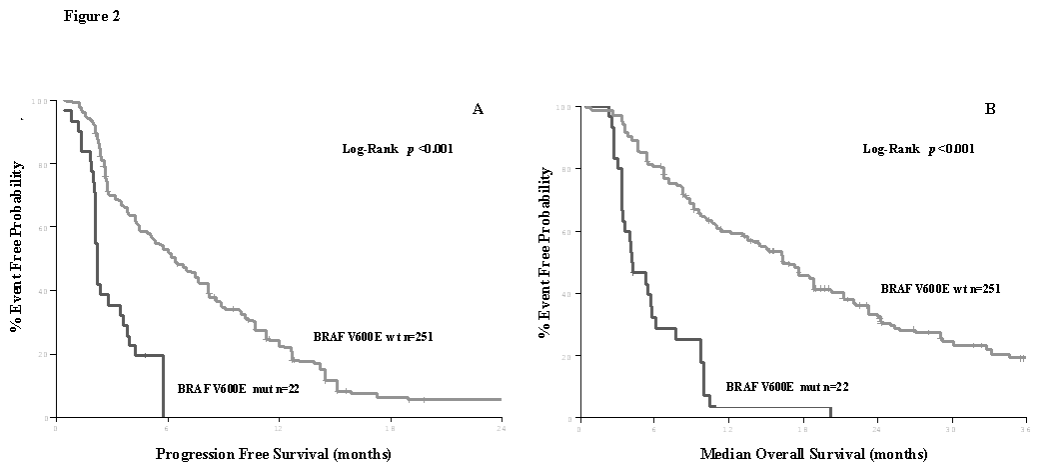
Progression Free Survival to salvage treatment with an anti-EGFR monoclonal (stratified for the treatment line) antibody according to *BRAF* ^*V600E*^mutation in 273 patients with metastatic Colorectal Cancer (A) and Median Overall Survival to salvage treatmentwith an anti-EGFR monoclonal antibody (stratified for the treatment line) according to *BRAF* ^*V600E*^mutation in 273 patients with metastatic Colorectal Cancer (B).

Seventy-four (25%) patients, with *KRAS* wt primary tumοrs, received an anti-EGFR monoclonal antibody in combination with chemotherapy as first line treatment. *BRAF* mutations were detected in 6 (8.1%) patients. Although the median PFS and OS were arithmeticaly lower in patients with BRAFV600E mutations compared to patients with wild type BRAFV600E status (4.2 vs. 11.1 months and 14.3 vs. 35.0 months, respectively) these differences were not statistically different probably because of the small sample size.. On the other hand, 273 patients with *KRAS* wt primary tumors were treated with an anti-EGFR mAb as second (84 patients, 31%) or subsequent line of treatment (189 patients, 69%). Patients with *BRAF*V600E mutant tumors (22 patients, 8%), presented significantly shortened PFS (2.2 vs. 6.0 months, *p*<0.0001) and mOS (4.3 vs. 17.4 months, *p*<0.0001) compared with those with *BRAF*V600E wt tumors (Figure 2A and B), stratified for the line of treatment. Another 13 patients with *BRAF* ^*V600E*^ mutation were not treated with anti-EGFR mAbs, in the 2^nd^ or higher treatment lines. Eleven of them received 2^nd^ line combination chemotherapy and the median PFS was 2.4 months while the median OS was 4.8, comparable with those observed in patients with *BRAF* ^*V600E*^ mutations treated with anti-EGFR mAbs.

## Discussion

In this present study we evaluated the impact of *BRAF*V600E testing for mCRC patients in daily clinical practice. To the best of our knowledge this is the largest prospective series of patients ever reported in the literature providing valuable data regarding epidemiological patterns, as well as, the impact of the *BRAF*V600E mutation status on patients’ outcome. Indeed the incidence of the mutation is significantly higher in patients with ECOG PS 2 (41%) compared to those with ECOG PS 0-1 (4.6%; *p*<0.001), but also in patients with undifferentiated tumors (14.1%), multi-metastatic disease (9.9%) and advanced age (10.8%) in comparison to those with differentiated tumors (4.3%; *p*=0.001), disease confounded in one metastatic site (3.6%; *p*=0.002) and aged ≤ 65 years (5.9%; *p*=0.004). On the other hand, the incidence of the *BRAF*V600E mutation was very low in patients with metastatic rectal cancer (2.8%). These data indicate that the *BRAFV600E* mutation is correlated with other know detrimental clinical prognostic factors whose may influence the patients’ outcome. On the other hand, one may argue that the aggressive biological behavior of tumors harboring a *BRAFV600E* is responsible for the presence of these clinical factors such as rapidly progressive multimetastatic disease and poor performance status or low probability of a secondary metastasectomy. Our study proposes that the assessment of *BRAFV600E* mutation is a step forward into “personalized treatment” since may modify the treatment intent (palliative or curative) and by that may influence the treatment strategy. 

Also, the current study, confirms in a prospectively analyzed patients’ cohort the adverse prognostic significance of the *BRAF*V600E mutation which has been previously reported in retrospective studies[3,6,8,11,14]. In fact, patients with *BRAF*V600E mutation in their primary tumor had significantly lower median PFS (4.1 vs. 11.6 months; HR: 4.07, 95% CI: 2.66-6.20; *p*<0.001) and OS (14.0 vs. 34.6 months; HR: 5.43, 95% CI: 3.60-8.18; *p*<0.001; Figure 1A-B), while the *BRAF*V600E mutation was revealed as the strongest independent factor for decreased PFS (HR: 4.1, 95% CI: 2.7-6.2; *p*<0.001) and OS (HR: 5.9, 95% CI: 3.7-9.5; *p*<0.001; Table 4). These findings are in agreement with previous retrospective studies from our group[8,11] and others[3,6,14,18] regarding the adverse prognostic significance of the *BRAF*V600E mutation in CRC. 

We also observed, in accordance with previous reports[3,7,8,16,18], that patients with *BRAF*V600E gain a limited if any benefit from the treatment anti-EGFR mAbs. In fact, patients with *BRAF*V600E mutant tumors treated with an anti-EGFR mAB in the second or subsequent line, presented significantly decreased median PFS (2.2 vs. 6.0 months, *p*<0.0001) and mOS (4.3 vs. 17.4 months, *p*<0.0001) compared to those with *BRAF*V600E wt tumors (Figure 2A and B). The same finding has been observed both in retrospective studies in series of patients[3,7,8,16,21], as well as, in randomized clinical trials[18]. On the other hand, the investigators in the CRYSTAL study reported a minor benefit from the addition of cetuximab to chemotherapy (FOLFIRI) in patients with *BRAF*V600E mCRC, but this finding remains questionable since an interaction test is not provided[19]. However, the adverse prognostic significance of the *BRAF*V600E mutation is clearly demonstrated even in this retrospective analysis of the CRYSTAL trial[19].. Our data as well as those from the studies previously mentioned suggest that the anti-EGFR moAbs are not capable to reverse the adverse prognosis of the *BRAFV600E* mutation. The current study is not capable to answer the questions where the *BRAFV600E* mutation has a predictive value for the treatment with anti-EGFR moAbs or if the patients with *BRAFV600E* mutation should be treated or not with anti-EGFR mAbs. This question should be probable addressed in a prospective manner either with a combination of BRAF and anti-EGFR inhibitor using an adaptive model or in a randomize trial using the *BRAFV600E* mutation as stratification factor.

Nevertheless, the analysis of mutations in RAS/RAF pathway has been proven significantly important for the management of patients with mCRC. In the present study patients with double WT type tumors present significantly higher PFS (HR: 1.89; *p*=0.034) and median OS (HR: 2.28; *p*=0.016) in comparison with those with a mutation in either of *KRAS* or *BRAF* genes. In addition, recently reported studies emphasize the importance of *KRAS* mutations outside hotspots in codon 12 and 13, as well as, of the *NRAS* mutations, especially in patients treated with Panitumumab [22], All the data emphasize the importance of the testing for RAS/RAF family in mCRC in order to design the optimal treatment strategy in the daily clinical practice. 

From the biological point of view our results support the concept that CRC with *BRAF*V600E is a distinct subset of the disease with specific biological characteristics. Indeed, the *BRAF*V600E mutation in CRC is correlated with MSI-H status and cyclin D1 overexpression, and characterizes a subgroup of patients with poor prognosis[9-11]. In addition, the distinct natural history and unresponsiveness of *BRAF*-mutant tumors to the commonly used chemotherapeutic regimens implies that *BRAF*V600E mutation does not simply substitute for *KRAS* activation in a linear signaling pathway but likely confers additional or distinct properties. For example, in cell cultures, the V600E mutation increases BRAF activity independent of *KRAS* and shows lower transforming activity[1], while inhibition of MEK with small molecules prevents tumor growth in *BRAF*-mutant tumor xenografts but not in *KRAS*-mutant counterparts[23]. These dissimilarities may in part explain the differences regarding the prognostic value of activating *KRAS* and *BRAF* mutations.

Finally, the analysis of the *BRAF*V600E in the daily clinical practice is feasible since it may be performed from the same DNA used for the analysis of the *KRAS* mutations which is mandatory for all patients with mCRC[24]. The analysis of the *BRAF*V600E using the allelic discrimination method is sensitive (sensitivity > 95%)[11,20], inexpesinsive [20] and provides the results within two hours. 

In summary, the *BRAF*V600E mutation identifies a subgroup of mCRC patients with distinct biological behavior, clinical characteristics and pathological features. These patients often present metastatic disease in multiple sites, have poor PS and a poor prognosis, being resistant to all currently available treatment options. The analysis of the *BRAF*V600E mutation in the daily clinical practice may be a step forward in the concept of “personalized” management of patients with mCRC, since new agents targeting this specific mutation are urgently warranted.
